# Soft and Conductive Polyethylene Glycol Hydrogel Electrodes for Electrocardiogram Monitoring

**DOI:** 10.3390/gels9120957

**Published:** 2023-12-06

**Authors:** Dongik Lee, Jihyang Song, Jungwoo Kim, Jaebeom Lee, Donghee Son, Mikyung Shin

**Affiliations:** 1 Department of Intelligent Precision Healthcare Convergence, Sungkyunkwan University (SKKU), Suwon 16419, Republic of Korea; dzzikno1@naver.com (D.L.); wjddn1998@naver.com (J.K.); jblee0226@gmail.com (J.L.); 2 Department of Artificial Intelligence System Engineering, Sungkyunkwan University (SKKU), Suwon 16419, Republic of Korea; sszang0210@gmail.com; 3 Department of Electrical and Computer Engineering, Sungkyunkwan University (SKKU), Suwon 16419, Republic of Korea; 4 Department of Biomedical Engineering, Sungkyunkwan University (SKKU), Suwon 16419, Republic of Korea

**Keywords:** conductive hydrogel, one-step process, electrode, electrocardiogram

## Abstract

The measurement of biosignals in the clinical and healthcare fields is fundamental; however, conventional electrodes pose challenges such as incomplete skin contact and skin-related issues, hindering accurate biosignal measurement. To address these challenges, conductive hydrogels, which are valuable owing to their biocompatibility and flexibility, have been widely developed and explored for electrode applications. In this study, we fabricated a conductive hydrogel by mixing polyethylene glycol diacrylate (PEGDA) with poly(3,4-ethylenedioxythiophene):polystyrene sulfonate (PEDOT:PSS) polymers dissolved in deionized water, followed by light-triggered crosslinking. Notably, this study pioneered the use of a PEGDA−PEDOT:PSS hydrogel for electrocardiogram (ECG) monitoring- a type of biosignal. The resulting PEGDA−PEDOT:PSS hydrogel demonstrated remarkable conductivity while closely approximating the modulus of skin elasticity. Additionally, it demonstrated biocompatibility and a high signal-to-noise ratio in the waveforms. This study confirmed the exceptional suitability of the PEGDA−PEDOT:PSS hydrogel for accurate biosignal measurements with potential applications in various wearable devices designed for biosignal monitoring.

## 1. Introduction

In the rapidly advancing field of current biomedical technology, the development of conductive hydrogels is emerging as an innovative technology, particularly gaining attention in the field of biosignal monitoring, for example, electrocardiography (ECG), electromyography (EMG) and electroencephalography (EEG) [1,2]. Biosignal monitoring is employed to acquire crucial medical information for disease diagnosis, health status monitoring, physical activity tracking, and the recording of biosignals. Conventional medical electrode devices and patches for biosignal monitoring typically includes electrodes, cable connectors, and an adhesive component [3]. Electrodes crafted with polarizable metals, including platinum and gold, or non-polarizable materials, such as Ag/AgCl, have traditionally set the benchmark due to their high conductivity; nevertheless, they have several drawbacks [4]. First, metal electrodes with excellent electrical conductivity have been conventionally adopted for neural recording but are less effective in surface ECG applications because of their potential for allergic reactions to metals, unstable contact causing motion artifacts, and the requirement for sharp tissue penetration [5,6,7]. Second, according to clinical reports regarding the patients’ discomfort after applying the Ag/AgCl-gel electrodes onto their skin, the silver-based electrodes caused skin irritations, such as rashes and itching, during electrophysiological monitoring [8,9,10,11,12,13]. Third, these conventional electrodes lack flexibility, making it challenging to maintain extremely conformal contacts with the soft and curved surface of the skin, which impedes the accurate measurement of biosignals [14,15].

In this context, conductive hydrogels offer innovative potential to overcome these issues. They provide perfect contact with the skin surface due to their flexibility, enabling precise and reliable signal measurements with high biocompatibility [16,17]. Fundamentally, hydrogels are three-dimensional networks of hydrophilic polymer chains with abundant water, which has tissue-like mechanical modulus [18,19,20]. Conductive hydrogels gain conductivity by adding conductive polymers or materials, in addition to their hydrogel characteristics [21]. Thus, conductive hydrogels can transmit electrical signals while maintaining their inherent properties such as flexibility and biocompatibility [22,23]. The integration of these characteristics renders conductive hydrogels exceptionally favorable for diverse applications, such as flexible wearable sensors, soft electronics, biomedicine, drug release devices, and drug delivery systems [24,25,26,27,28,29,30,31,32,33].

**Figure 1:**
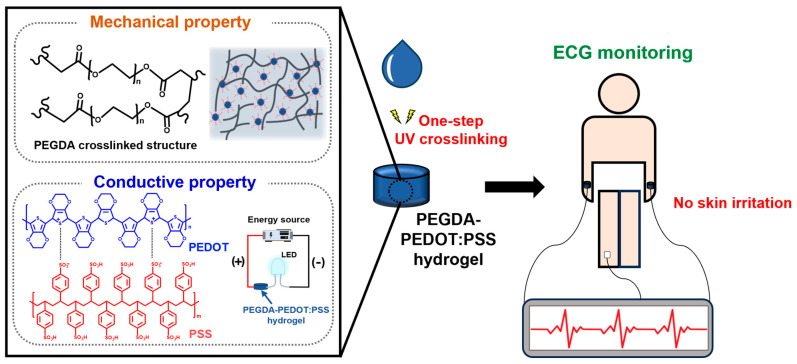
Comprehensive illustration of the PEGDA–PEDOT:PSS conductive hydrogel for ECG monitoring. PEG, PEDOT and PSS are represented with black, blue and red text, respectively, along with their chemical structures.

In this study, we present a poly(ethylene glycol) diacrylate (PEGDA) –poly(3,4-ethylenedioxythiophene):poly(styrene sulfonate) (PEDOT:PSS) conductive hydrogel, achieved by blending PEDOT:PSS as a conducting polymer with PEGDA as a hydrogel material for electrocardiogram (ECG) monitoring, as part of biosignal analysis (Figure 1). PEDOT: PSS, a polythiophene derivative, stands out as a highly promising conductive polymers owing to its exceptional chemical stability, electrical conductivity, and biocompatibility [34,35]. Additionally, PEGDA hydrogels have been synthesized by cross-linking PEG diacrylate monomers, resulting in a three-dimensional polymer network capable of retaining substantial amounts of water [36]. Because of their biocompatibility and capability to mimic the extracellular matrix (ECM) present in living tissues, PEGDA hydrogels have diverse applications in various biomedical fields [37].

Ultimately, the PEGDA–PEDOT:PSS hydrogel developed in this study has unique significance for several reasons. First, although previous studies have focused on the development of conductive hydrogels using PEGDA and PEDOT:PSS, our study is the first attempt at biosignal monitoring, highlighting its distinctive importance. In addition, we manufactured this hydrogel using a ‘one-step process’ that involved the simple mixing of PEGDA and PEDOT:PSS. This one-step process is a crucial feature that can significantly reduce production costs and shorten the production time. This efficiency has the potential for large-scale production, facilitating the commercialization of this technology. Furthermore, this hydrogel has the potential for application as a wearable electrode for monitoring various biosignals as well as ECG, while highlighting promising technological advancements in patient care and health monitoring.

## 2. Results and Discussion

### 2.1. Characteriazation of PEGDA–PEDOT:PSS Hydrogel

**Figure 2:**
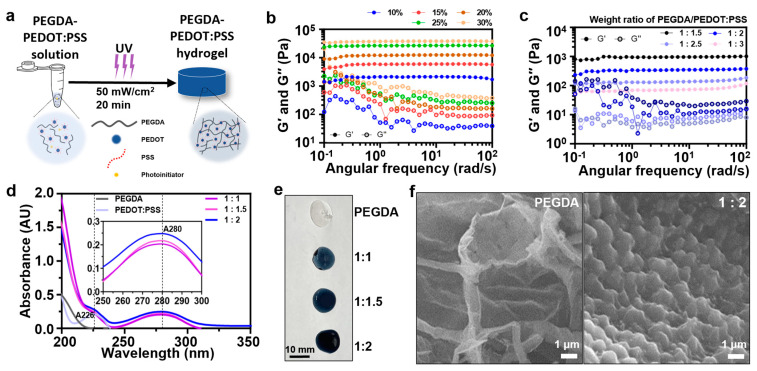
Characterization of PEGDA–PEDOT:PSS hydrogel. (**a**) Illustration of the PEGDA–PEDOT:PSS hydrogel fabrication process. (**b**,**c**) Oscillation frequency sweep measurements of PEGDA–PEDOT:PSS hydrogel for different *w*/*v* (%) of the PEGDA–PEDOT:PSS mixture (**b**), and for different weight ratios of the PEGDA to PEDOT:PSS at a concentration of 10 *w*/*v* % in the total solution volume (**c**). Solid circles depict storage modulus (G′), while open circles represent loss modulus (G″). (**d**) UV-Vis spectra of PEGDA (dark gray), PEDOT:PSS (light violet), and PEGDA–PEDOT:PSS mixture (blue, pink, and purple). An absorbance reading at the wavelength of 226 nm (A_226_) indicates PEDOT:PSS, and the absorbance at 280 nm (A_280_) means the existence of PEDOT:PSS in each PEGDA–PEDOT:PSS mixture. (**e**) Photograph images of PEGDA–PEDOT:PSS hydrogels. (**f**) FE-SEM images of PEGDA hydrogel (**left**) and PEGDA–PEDOT:PSS hydrogel (**right**). The indicated ratio in this section represents the weight ratio of PEGDA to PEDOT:PSS at a concentration of 10 *w*/*v* % of the total solution volume.

The mechanical disparity between tissue-interfacing materials can result in not only decreased effectiveness of biosignal recording but also immunological responses in the contacted region [38,39,40]. Hence, there is a significant need for materials that can be intentionally engineered to exhibit mechanical characteristics resembling those of tissues while preserving their electronic functionality [41]. Therefore, it is essential that the mechanical properties of the PEGDA–PEDOT:PSS hydrogel closely resemble those of the skin. According to Holt, B. et al. (2008), the properties of the whole skin (epidermis and dermis) exhibit a storage modulus (G′) within a range of 0.1 to 1 kPa under a frequency sweep of 1 to 10 rad/s and a 0.5% strain [42]. Therefore, we aimed to create a hydrogel possessing comparable characteristics within a similar range. The process for preparing the PEGDA–PEDOT:PSS hydrogel is shown in Figure 2a. This is a straightforward one-step procedure that involves mixing PEGDA and PEDOT:PSS solutions, adding deionized water (DW) and Irgacure 2959, a type I photoinitiator (at a concentration of 0.1% of the total solution volume), and subsequently exposing it to ultraviolet (UV) radiation at an intensity of 50 mW/cm^2^ for 20 min.

Rheometric measurements were performed on the fabricated PEGDA–PEDOT:PSS hydrogels. Initially, the weight ratio of PEGDA to PEDOT:PSS was fixed at 1:1, and the weight/volume (*w*/*v*) percentage (%) of the mixed solution was adjusted to 10, 15, 20, 25, and 30 *w*/*v* % of the total solution (Figure 2b). When PEGDA and PEDOT:PSS constituted 10 *w*/*v* % of the total solution, the average G′ was 2.01 kPa. For the remaining concentrations of 15, 20, 25, and 30 *w*/*v* %, the average G′ were 5.74, 11.97, 26.56, and 37.65 kPa, respectively. Therefore, the skin-like modulus was best approximated when PEGDA and PEDOT:PSS were mixed in a 1:1 weight ratio, a concentration of 10 *w*/*v* %.

Based on a previous report that a small quantity of PEDOT:PSS enhances the photocrosslinking kinetics [43], we assumed that increasing of PEDOT:PSS would result in a decrease in G′ of the PEGDA–PEDOT:PSS hydrogels. The weight ratio of PEGDA to PEDOT:PSS was then incremented from 1:1 to 1:3, with G′ measurements presented in Figure 2c. As expected, G′ was decreased as the PEDOT:PSS content increased. The results indicated that at a 1:2 of PEGDA to PEDOT:PSS, the storage modulus (average of G’ = 0.34 kPa) was observed within a range of 0.1 to 1 kPa, aligning with the desired skin-like properties. Consequently, the optimal hydrogel composition was determined to be a 1:2 weight ratio of PEGDA to PEDOT:PSS at a concentration of 10 *w*/*v* % of the total solution volume.

UV absorbance measurements were conducted to validate the increase in PEDOT:PSS content (Figure 2d). Owing to the limitations of measuring the absorbance of the hydrogels using UV spectroscopy, the PEGDA–PEDOT:PSS solutions were diluted with DW to a concentration of 0.05%. These results confirmed that a higher PEDOT:PSS content led to increased absorbance. In addition, the absence of a new peak indicated that no chemical bonding occurred between PEGDA and PEDOT:PSS. In Figure 2e, the images of the PEGDA–PEDOT:PSS hydrogels demonstrate that as the weight ratio of the PEDOT:PSS increased, the color darkened, providing visual confirmation. Additionally, the cross-sectional morphology of each hydrogel was examined (Figure 2f). Field emission scanning electron microscopy (FE-SEM) images of the PEGDA–PEDOT:PSS hydrogel showed a uniform distribution of PEDOT:PSS particles, whereas the pristine PEGDA hydrogel featured a network of pore walls but lacked PEDOT:PSS particles. In all subsequent experiments following this section, the PEGDA–PEDOT:PSS hydrogel had a PEGDA-to-PEDOT:PSS ratio of 1:2 at 10 *w*/*v* % of the total solution volume.

### 2.2. Electrical Characterization of PEGDA–PEDOT:PSS Hydrogel

**Figure 3:**
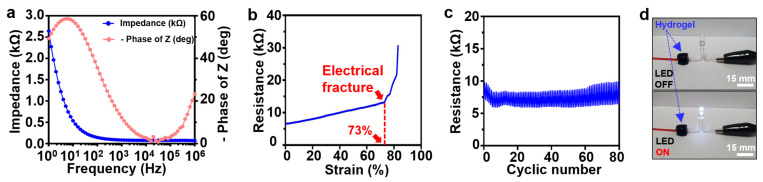
Electrical characterization of PEGDA–PEDOT:PSS hydrogel. (**a**) Impedance of PEGDA–PEDOT:PSS hydrogel (blue) and phase angle (light red). (**b**) Resistance of PEGDA–PEDOT:PSS hydrogel to increasing tensile strain at a speed of 15 mm/min. (**c**) Cyclic stretching test between 0% and 50% repetitively for strains at a speed of 30 mm/min at 80 times. (**d**) LED emission in an electrical circuit connected to PEGDA–PEDOT:PSS hydrogel (blue arrow).

To validate the efficacy of the PEGDA–PEDOT:PSS hydrogel as a conductive hydrogel, its electrical performance was measured. Initially, we measured the impedance of the PEGDA–PEDOT:PSS hydrogel over a range of frequencies to assess its suitability for efficient monitoring of ECG signals (Figure 3a) [44]. In contrast to the pure electrical conductivity observed in metallic components, conductive hydrogels exhibit a combination of electrical and ionic conductivity [1], leading to consistently minimal impedance values across various frequency ranges [45]. Specifically, our PEGDA–PEDOT:PSS hydrogel demonstrated a low impedance of 2.64 kΩ at 1 Hz. At 10 Hz, the impedance measures almost 0.5 kΩ, indicating a remarkably low impedance level. In addition, the phase angle remained close to 0° within the frequency range of 10^3^–10^5^ Hz. Bioelectronics attached to the skin should maintain their electrical performance even under strains of at least 30%, as stretching of the skin caused by human movement is approximately 30% [46,47]. Therefore, we investigated the electrical properties of PEGDA–PEDOT:PSS hydrogels in response to stretching. Notably, the hydrogel was still able to maintain an electrical conduction value of up to 73% of the tensile strain, suggesting its potential to sustain a stable electrical performance even during skin deformations resulting from movement (Figure 3b). Furthermore, we confirmed that the electrode maintained a stable electrical performance during 80 cycles of repetitive mechanical deformation between 0 and 50% strain (Figure 3c). These results indicate the potential applications of the PEGDA–PEDOT:PSS hydrogel for monitoring biosignals. Finally, the conductive properties of the PEGDA–PEDOT:PSS hydrogel were examined by employing using light-emitting diodes (LEDs) (Figure 3d). We followed the approach outlined in a previous report from Kim, S. et al. (2023) [48]. One terminal of the LED was linked to the energy source, while the opposite end was attached to the PEGDA–PEDOT:PSS hydrogel and linked to the energy source to illuminate the LED. The PEGDA–PEDOT:PSS hydrogel functioned effectively as an electrode, enabling LED illumination. The results presented in this section demonstrate the outstanding electrical performance of the PEGDA–PEDOT:PSS hydrogel and suggest its potential utility in flexible electronics, particularly in bioelectronics.

### 2.3. Biological Safety and Swelling Behavior of PEGDA–PEDOT:PSS Hydrogel

**Figure 4:**
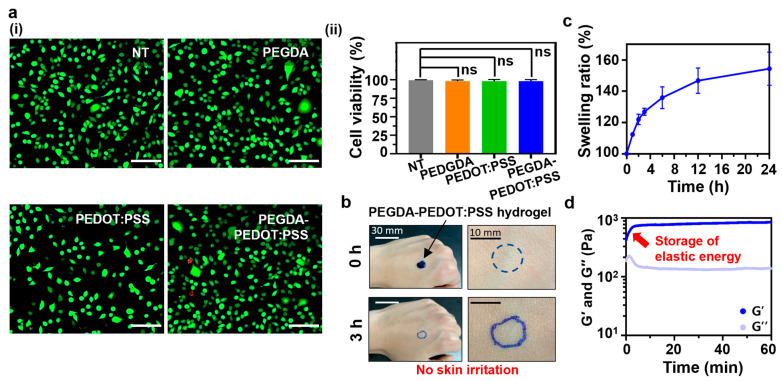
Biological safety and swelling behavior of PEGDA–PEDOT:PSS hydrogel for biomedical application. (**a**): (**i**) Fluorescent images of L929 cells at 24 h after the treatment of the PEGDA hydrogel, PEDOT:PSS film, PEGDA–PEDOT:PSS hydrogel. Green dots for live cells and red dots for dead cells. ‘NT’ means none of treatment. (Scale bar = 100 μm); (**ii**) Quantitative analysis of the cell viability (%) (*n* = 3). One-way ANOVA. ‘ns’ represents ‘not significant’. (**b**) Skin allergy tests of PEGDA–PEDOT:PSS hydrogel after 3 h placement on the skin. Top photos showing the skin at 0-h and bottom photos at 3 h. (**c**) Swelling characteristic of PEGDA–PEDOT:PSS hydrogels at specific time intervals (*n* = 5). (**d**) Water reabsorption capability of PEGDA–PEDOT:PSS hydrogel in DW for 60 min. Blue line and light purple line indicate storage modulus (G′) and loss modulus (G″), respectively. A red arrow shows increase of the elastic energy within the hydrogels by water absorption.

The PEGDA–PEDOT:PSS hydrogel holds promise for a diverse range of bioelectronics in biomedical applications. Therefore, in vitro, we assessed the biocompatibility of the hydrogel via mouse fibroblast cells (L929) (Figure 4a). After treating the cell cultures with eluates from the hydrogels (PEGDA and PEGDA–PEDOT:PSS) or pure PEDOT:PSS film, it was observed that approximately 98% of the cells in all test groups remained viable. These results aligned with those of previous studies on the cytotoxicity of PEGDA and PEDOT:PSS, confirming the excellent biocompatibility of the PEGDA–PEDOT:PSS hydrogel [49,50]. To ensure the safety of the PEGDA–PEDOT:PSS hydrogel, we conducted skin allergy tests on a human arm (Figure 4b). The PEGDA–PEDOT:PSS hydrogel did not cause any skin irritation such as redness or swelling. These findings indicate that the PEGDA–PEDOT:PSS hydrogel is non-cytotoxic and can be safely applied directly to skin tissue. Swelling-resistant properties are crucial for hydrogels because excessive swelling resulting from changes in osmotic pressure or physiological conditions can significantly impede their practical utility by compromising both their mechanical and electrical performances [51]. Therefore, precise control of the swelling ratio is essential to ensure performance stability and has substantial practical significance [52]. In this context, we examined the swelling kinetics of the PEGDA–PEDOT:PSS hydrogel immersed in phosphate-buffered saline (PBS) (Figure 4c). After approximately 1 h, the weight had increased by less than 1.2 times. Over the course of 12 h, there was a slight increase, reaching just over 1.4 times. Even after 24 h, the weight gain remained below 1.6 times. This suggests that the hydrogel experienced minimal swelling within this timeframe. Following the investigation into the swelling kinetics, we examined the reabsorption capability of the PEGDA–PEDOT:PSS hydrogel (Figure 4d). The G′ values initially increased during the early stages of water absorption (red arrow) but soon reached an extremely slight and slow increase. This initial rise in G′ was attributed to the storage of elastic energy. As water molecules diffused into the freeze-dried PEGDA–PEDOT:PSS hydrogel, the cross-linkage junctions within the hydrogel networks dispersed, facilitating volumetric expansion and the subsequent storage of elastic energy which is proportional to mechanical modulus (e.g., G′) [53]. The phase of slow increase of G′ indicates a state where few water molecules diffused into the hydrogel.

### 2.4. ECG Monitoring Using PEGDA–PEDOT:PSS Hydrogel

**Figure 5:**
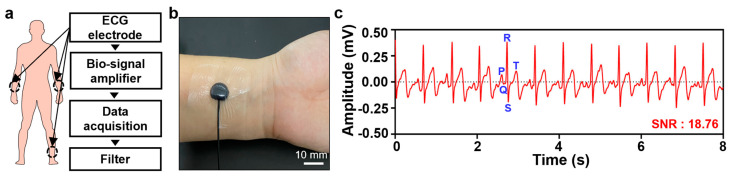
ECG measurement using PEGDA–PEDOT:PSS hydrogel. (**a**) Acquisition and filtering process of ECG signal data with PEGDA–PEDOT:PSS hydrogels (right and left wrist) and a commercial electrode (right ankle). (**b**) Photograph of PEGDA–PEDOT:PSS hydrogel attached to the wrist, secured with Tegaderm film to hold both hydrogel and electrical wire in place. (**c**) P–Q–R–S–T waves in ECG signals with an SNR of 18.76.

Finally, we evaluated the applicability of the PEGDA–PEDOT:PSS hydrogel in monitoring ECG signals. We affixed the PEGDA–PEDOT:PSS hydrogels to the left and right wrists, and a commercial ECG electrode to the right ankle for ECG recording (Figure 5b). Figure 5a illustrates the process of obtaining ECG signal data using the electrodes. The acquired ECG signals exhibited distinct P–Q–R–S–T wave patterns, which are characteristic of clinical ECGs (Figure 5c). Moreover, the ECG data exhibited a signal-to-noise ratio (SNR) of 18.76, indicating a substantial reduction in the artifacts. These findings confirm the suitability of the PEGDA–PEDOT:PSS hydrogel as an electrode for monitoring or recording biosignals.

## 3. Conclusions

We developed a PEGDA–PEDOT:PSS conductive hydrogel with mechanical properties that closely mimic those of human skin. This was achieved by blending PEGDA and PEDOT:PSS, and subjecting them to UV exposure. Although previous studies have explored the development of conductive hydrogels using PEGDA and PEDOT:PSS, this research is the first attempt at biosignal monitoring, underlining its unique significance. The key challenge in our study was to maintain the crosslinking of PEGDA while increasing the proportion of PEDOT:PSS, thereby establishing the mechanical properties and optimizing the electrical performance. Consequently, the PEGDA–PEDOT:PSS hydrogel exhibited a relatively low impedance and maintained consistent resistance levels even under tensile strain. Moreover, cyclic stretching tests confirmed the resistance stability, suggesting its potential to maintain reliable electrical performance during skin movement. We validated its electrical performance by employing a PEGDA–PEDOT:PSS hydrogel as an electrode to power an LED connected to an energy source. Additionally, the hydrogel exhibited exceptional biocompatibility and ECG signals with a high SNR. These attributes emphasize the versatility of the hydrogel for application in various wearable devices designed for precise biosignal monitoring. We anticipate that future research will expand its applicability to the accurate measurement of a wide range of biosignals in the biomedical field, extending beyond ECG monitoring.

## 4. Materials and Methods

### 4.1. Materials

Poly(3,4-ethylenedioxythiophene):poly(styrenesulfonate) (PEDOT:PSS) solution (1.0–1.3% solid content, Clevios™ PH1000) was purchased from Heraeus Group (Hanau, Germany). Poly(ethylene glycol) diacrylate (PEGDA, Molecular weight = 4 kDa) and 2-hydroxy-4′-(2-hydroxy-ethoxy)2-methyl propiophenone (Irgacure 2959), and dimethyl sulfoxide (DMSO) were purchased from Sigma-Aldrich (St. Louis, MO, USA). Irgacure 2959 was used as a type I photoinitiator during ultraviolet (UV) irradiation. Phosphate-buffered saline (PBS, 10× pH 7.2) was purchased from Welgene (Gyeongsan, Republic of Korea).

### 4.2. Fabrication of PEGDA Hydrogel, PEGDA–PEDOT:PSS Hydrogel and Pure PEDOT:PSS Film

#### 4.2.1. PEGDA Hydrogel

PEGDA was added to deionized water (DW), containing 0.1% Irgacure 2959 at a concentration of 10% *w*/*v* of the total volume. Subsequently, the mixture was exposed to UV irradiation (OmniCure^®^ S1500 Spot UV Curing system, Excelitas technologies, Waltham, MA, USA) at an intensity of 50 mW/cm^2^ for 20 min to fabricate the PEGDA hydrogel.

#### 4.2.2. PEGDA–PEDOT:PSS Hydrogel

The *w*/*v* (%) of the PEGDA and PEDOT:PSS mixed solutions was determined as follows. PEGDA–PEDOT:PSS solutions were prepared by blending different weight percentages of PEGDA (10, 15, 20, 25, and 30 *w*/*v* %) with the PEDOT:PSS solution at a 1:1 weight ratio within the range of the final solution. DW and Irgacure 2959 were then added to make the final volume. Irgacure 2959 accounts for 0.1% of the total volume. Each mixture was exposed to UV irradiation for 20 min at an intensity of 50 mW/cm^2^, resulting in the fabrication of PEGDA–PEDOT:PSS hydrogels.

Subsequently, PEGDA–PEDOT:PSS solutions were prepared using various weight ratios (1:1, 1:1.5, 1:2, 1:2.5, and 1:3) of PEGDA to PEDOT:PSS at 10 *w*/*v* % of the total solution volume. DW and Irgacure 2959 were used to achieve the desired total volume. Irgacure 2959 accounts for 0.1% of the total volume. Each mixture was then exposed to UV irradiation for 20 min at an intensity of 50 mW/cm^2^, leading to the fabrication of the PEGDA–PEDOT:PSS hydrogels. The final composition of the PEGDA–PEDOT:PSS hydrogel had a PEGDA-to-PEDOT:PSS ratio of 1:2 at 10 *w*/*v* % of the total solution volume, therefore, all PEGDA–PEDOT:PSS hydrogels used in experiments following Figure 2 were prepared under this composition condition.

#### 4.2.3. Pure PEDOT:PSS Film

The method of Lu, B. et al. (2019) was used as a reference [54], and a slightly modified approach was employed to create a pure PEDOT:PSS film. The PEDOT:PSS solution was mixed with DMSO in a 3:17 ratio. Upon further stirring for 24 h at room temperature, the mixed solution was solvent-cast directly onto a plastic cryomold (15 mm × 15 mm) and dried at 60 °C for 24 h.

### 4.3. Rheological Characterization of PEGDA–PEDOT:PSS Hydrogels

The rheological characteristics of the PEGDA–PEDOT:PSS hydrogels were assessed via oscillation frequency sweep tests performed using a Discovery Hybrid Rheometer 2 (TA Instruments, New Castle, DE, USA). Rheological assessments were performed using a 20 mm parallel-plate setup, where the gap size was set at 200 μm. The storage modulus (G′) and loss modulus (G″) were determined at room temperature under oscillation frequency sweeps (0.1–100 rad/s) with a 0.5% strain applied.

### 4.4. UV-Vis Characterization of PEGDA–PEDOT:PSS Hydrogels

The difference in the ratio of PEGDA to PEDOT:PSS was verified using a UV-Vis spectrometer (Agilent 8453, Agilent Technologies, Santa Clara, CA, USA). However, because of the limitations in measuring the absorbance of hydrogels containing 10 *w*/*v* % PEGDA–PEDOT:PSS, we performed UV-Vis measurements after diluting each pre-UV-irradiated solution with DW. Specifically, for PEGDA, we achieved a concentration of 0.01% by diluting with DW. Similarly, the remaining PEGDA–PEDOT:PSS and PEDOT:PSS solutions were diluted to 0.05% with DW. DW was used as a blank solution.

### 4.5. Morphological Analysis of PEGDA Hydrogel and PEGDA–PEDOT:PSS Hydrogel

To analyze the morphological characteristics of the PEGDA and PEGDA–PEDOT:PSS hydrogels (10 *w*/*v* %, 1:2), field-emission scanning electron microscopy (FE-SEM, JSM-7500F, JEOL, Tokyo, Japan) was used. The PEGDA and PEGDA–PEDOT:PSS hydrogels were lyophilized, cross-sectioned using a blade, and coated with iridium.

### 4.6. In Vitro Cytotoxicity Test

To evaluate the in vitro cytocompatibility of the hydrogels, mouse fibroblast cells (L929) were initially cultured in growth media composed of Dulbecco’s Modified Eagle Medium (DMEM, low glucose, Gibco, Waltham, MA, USA) supplemented with 10% (*v*/*v*) fetal bovine serum (FBS, Gibco, USA) and 1% (*v*/*v*) penicillin-streptomycin (Gibco, USA). The releasates from the PEGDA, PEDOT:PSS and PEGDA–PEDOT:PSS (100 mg) were collected in the medium (1 mL) for 24 h at 37 °C. When the cells reached 80% confluence, they were seeded into 24-well plates at a density of 2.5 × 10^4^ cells per well and incubated overnight in a 5% humidified CO_2_ incubator at 37 °C. After rinsing with Dulbecco’s Phosphate-Buffered Saline (DPBS), the medium containing the releasates was added to each well after a ten-fold dilution. They were then incubated overnight once more in a 5% humidified CO_2_ incubator at 37 °C. Afterwards, the cell viability was assessed using a Live/Dead Assay Kit (Thermo Fisher Scientific, Seoul, Republic of Korea). After rinsing with DPBS, the cells in each well were incubated in a working solution, which was composed of DPBS (0.5 mL), 2 mm of Ethidium homodimer-1 solution (1 µL), and 4 mm of Calcein AM solution (0.25 µL), for 1 h at 37 °C. Finally, live and dead cells were observed using fluorescence microscopy (DMi8, Leica, Wetzlar, Germany). The number of green dots representing live cells and red dots representing dead cells were quantified using ImageJ software (v. 1.8.0_345), and cell viability (%) was calculated as the ratio of live cells to the total number of cells.

### 4.7. Skin Allergic Reaction Test of PEGDA–PEDOT:PSS Hydrogels

To assess skin allergic reactions, a PEGDA-PEDOT hydrogel was applied to the human arm. After 3 h, the hydrogel was gently removed, and any skin color changes or swelling were observed. Institutional Review Board (IRB) approval (NO. SKKU 2023-11-019) for this study was obtained by the author from Sungkyunkwan University.

### 4.8. Swelling Studies of PEGDA–PEDOT:PSS Hydrogels

For investigation of the swelling kinetics of the PEGDA–PEDOT:PSS hydrogel, the hydrogels were immersed in a 1× phosphate-buffered saline (PBS). The 1× PBS solution was prepared by diluting a 10× PBS solution in a 1:10 ratio. Subsequently, at specific time intervals (0, 1, 2, 3, 6, 12, and 24 h), we determined the weight of each hydrogel after removing superficial moisture. Five identical PEGDA–PEDOT:PSS hydrogels were used in these experiments. The swelling ratio was calculated using the following equation:

$$Swelling\;ratio\left(\%\right)=\frac{W_{s}-W_{o}}{W_{o}}\times 100,$$

where *W_s_* and *W_o_* represent the weights of the swollen hydrogel at different times and the weight of the initial hydrogel, respectively.

For the investigation of water reabsorption kinetics in the PEGDA–PEDOT:PSS hydrogel, the hydrogel was freeze-dried for 4 h. Subsequently, the dried hydrogel was placed on a rheometer (Discovery Hybrid Rheometer 2, TA Instruments, New Castle, DE, USA), and DW was evenly sprayed over it. Rheological assessments were conducted using a 20 mm parallel-plate setup with a gap size set at 800 μm. The G′ and G″ were measured at 10 rad/s of fixed frequency and a 0.5% strain applied for 60 min. The amount of DW was applied in an amount 1.6 times the volume of the PEGDA–PEDOT:PSS hydrogel.

### 4.9. Electrical Characterization of PEGDA–PEDOT:PSS Hydrogels

#### 4.9.1. Impedance

The electrochemical impedance of the PEGDA–PEDOT:PSS hydrogel was measured using a potentiostat in the frequency range of 1–10^6^ Hz in 1× PBS solution at a scan rate of 100 mV/s. An Ag/AgCl reference electrode and a platinum counter electrode were used. To reduce the capacitance at the connector interface, we measured the PEGDA–PEDOT:PSS hydrogel electrode on gold by connecting wires to the gold.

#### 4.9.2. Resistance

To measure the electrical properties of the PEGDA–PEDOT:PSS hydrogel, all hydrogel electrodes were loaded at 5 mm × 5 mm and measured using a digital multimeter (Keithley 2450 Digital Multimeter, Clackamas, OR, USA) and an *x*-axis stretcher (SMC-100, Jaeil Optical Corp., Daegu, Republic of Korea). The samples were loaded using double-sided tape and instant adhesive. The samples were stretched at a rate of 15 mm/min for the strain resistance test, whereas for the cyclic test, they were repeatedly stretched for 80 cycles at a rate of 30 mm/min.

#### 4.9.3. LED Emission

To verify the conductive properties of the PEGDA–PEDOT:PSS hydrogel, the hydrogel and LED were connected in series and linked to a Keithley 2450 source meter (Tektronix Inc., Clackamas, OR, USA) as a power supply. The successful illumination of a Light-Emitting Diode (LED) was evidence of its conductive properties.

### 4.10. ECG Signals Monitoring

After affixing the PEGDA–PEDOT:PSS hydrogel electrodes to the skin, we secured both the electrodes and wires using a Tegaderm film (3M, Maplewood, MN, USA) for ECG measurements. The recorded signals were amplified using a biological signal amplifier (Bio Amp FE231, AD Instruments, Dunedin, New Zealand) and recorded using a data-collection device (PowerLab 8/35, AD Instruments). Data were acquired and filtered using LabChart 8 Pro software (AD Instruments, Bella Vista, New South Wales, Australia). Institutional Review Board (IRB) approval (NO. SKKU 2023-11-019) for this study was obtained by the author from Sungkyunkwan University.

### 4.11. Statistical Analysis

Statistical significance was determined through a one-way ANOVA followed by a post-hoc Tukey test. The data is presented as the mean ± standard deviation, and all experiments were conducted at least three times.

## Data Availability

The data presented in this study are available in the article.
